# Dissecting Functions of the Conserved Oligomeric Golgi Tethering Complex Using a Cell-Free Assay

**DOI:** 10.1111/tra.12128

**Published:** 2013-10-31

**Authors:** Nathanael P Cottam, Katherine M Wilson, Bobby G Ng, Christian Körner, Hudson H Freeze, Daniel Ungar

**Affiliations:** 1 Department of Biology, University of YorkYork, UK; 2 Genetic Disease Program, Sanford Children's Health Research Center, Sanford-Burnham Medical Research InstituteLa Jolla, CA, USA; 3 Department of Paediatrics, Medical University of HeidelbergHeidelberg, Germany

**Keywords:** cell-free reconstitution, congenital disorders of glycosylation, conserved oligomeric Golgi complex, glycosylation enzyme sorting, Golgi apparatus, vesicle tethering

## Abstract

Vesicle transport sorts proteins between compartments and is thereby responsible for generating the non-uniform protein distribution along the eukaryotic secretory and endocytic pathways. The mechanistic details of specific vesicle targeting are not yet well characterized at the molecular level. We have developed a cell-free assay that reconstitutes vesicle targeting utilizing the recycling of resident enzymes within the Golgi apparatus. The assay has physiological properties, and could be used to show that the two lobes of the conserved oligomeric Golgi tethering complex play antagonistic roles in *trans*-Golgi vesicle targeting. Moreover, we can show that the assay is sensitive to several different congenital defects that disrupt Golgi function and therefore cause glycosylation disorders. Consequently, this assay will allow mechanistic insight into the targeting step of vesicle transport at the Golgi, and could also be useful for characterizing some novel cases of congenital glycosylation disorders.

Vesicle transport is responsible for protein sorting within the eukaryotic secretory pathway. It can be divided into four principal stages: formation of the vesicle (budding); movement of the vesicle to the target membrane (transport); loose attachment of the vesicle to the target (tethering) and merger of the vesicle and target membranes (fusion). Of these stages, budding and fusion are the best characterized. In both cases, detailed mechanistic characterization has been greatly facilitated by the development of cell-free reconstitutions in the form of budding (1,2) and fusion (3,4) assays. Conversely, the lack of cell-free assays for other steps in the transport pathway has caused our mechanistic understanding of these processes to lag behind. In particular, we know very little about the molecular details of vesicle tethering. Earlier efforts for reconstituting the whole of vesicle transport (5–7) used an enzymatic reaction in the lumen of the target organelle to indicate successful cargo delivery. This assay has the disadvantage of monitoring a step far downstream of vesicle tethering. The large number of steps between the actual tethering process and the assay readout means that subtle changes in tethering efficiency, which have to be monitored for a detailed mechanistic description of the process, will likely go unnoticed in this assay. Indeed, only one study has used it for the mechanistic dissection of the vesicle targeting process in close to 30 years (8). More recently developed assays using fluorescent labelling of membranes do not reconstitute vesicle transport *per se*, but rather the transport and fusion of whole organelles (9,10), such as vacuoles or endosomes, and therefore do not recapitulate the sorting aspect of vesicle-based transport.

The posttranslational modification of secreted proteins is compartmentalized into several cisternae at the Golgi (11), and the enzymes performing the modifications use different retrograde transport vesicles to be sorted into their appropriate locations (12). The Golgi apparatus is therefore ideally suited for reconstituting vesicle transport and especially vesicle-based sorting (5). Isolated Golgi membranes retain their transport activity (5), and can be used in conjunction with transport vesicles to reconstitute the retrograde vesicular sorting of resident Golgi enzymes (13). The transport of these retrograde vesicles depends on several transport factors, with the conserved oligomeric Golgi (COG) complex playing a central role (14,15). COG is a hetero-octamer that combinatorially interacts with several Rab-type small GTPases and coiled coil tethers of the golgin family to accomplish vesicle tethering (16). It is therefore not surprising that COG defects cause aberrant glycosylation (17) because of the missorting of glycosylation enzymes (18).

A large proportion of the inherited glycosylation defects in humans are classed as congenital disorders of glycosylation (CDGs). CDGs are rare because they are very difficult to diagnose based on clinical symptoms alone, yet the cases diagnosed so far have arisen from defects in over 30 different genes, including genes involved in Golgi organization and trafficking (19,20). Because of a lack of suitable cellular assays, the defective gene in novel CDG cases is difficult to pin down, especially when Golgi organization rather than a specific glycosylation reaction is affected. The first described CDG patients with COG defects were siblings with a Cog7 defect (21), and since then CDG-causing mutations in six of the eight COG subunits (COGs 1–8) have been described (22). The most severe of these are the aforementioned Cog7 and a Cog6 mutation (23), both of which result in death within the first few months after birth. Most of the patient-derived mutations fall into the Cog5–8 part of the complex, also known as lobe B. Lobe B has been implicated in mediating the vesicular sorting of *trans*-Golgi enzymes (24,25), likely by its functional interactions with the tethering and fusion machinery in this Golgi region (26). Lobe A (Cog1–4), in contrast, plays a role in Golgi organization and *cis*-Golgi sorting (24,26), yet its potential contribution to *trans*-Golgi vesicular sorting has not been elucidated.

Here, we use the recycling of the late-Golgi enzyme β-1,4-galactosyltransferase I (GalT) (27) as the basis for the cell-free reconstitution of vesicle transport. Our system can be used to dissect the role of COG's different parts in *trans*-Golgi vesicle tethering. The data imply that lobe A is inhibitory for lobe B-mediated late-Golgi vesicle targeting. Moreover, the assay shows sensitivity to a novel patient-derived mutation opening up the possibility of its use for the cellular confirmation of CDG mutations that are defective in Golgi-enzyme trafficking.

## Results

### Development of the cell-free assay

**Figure 1:**
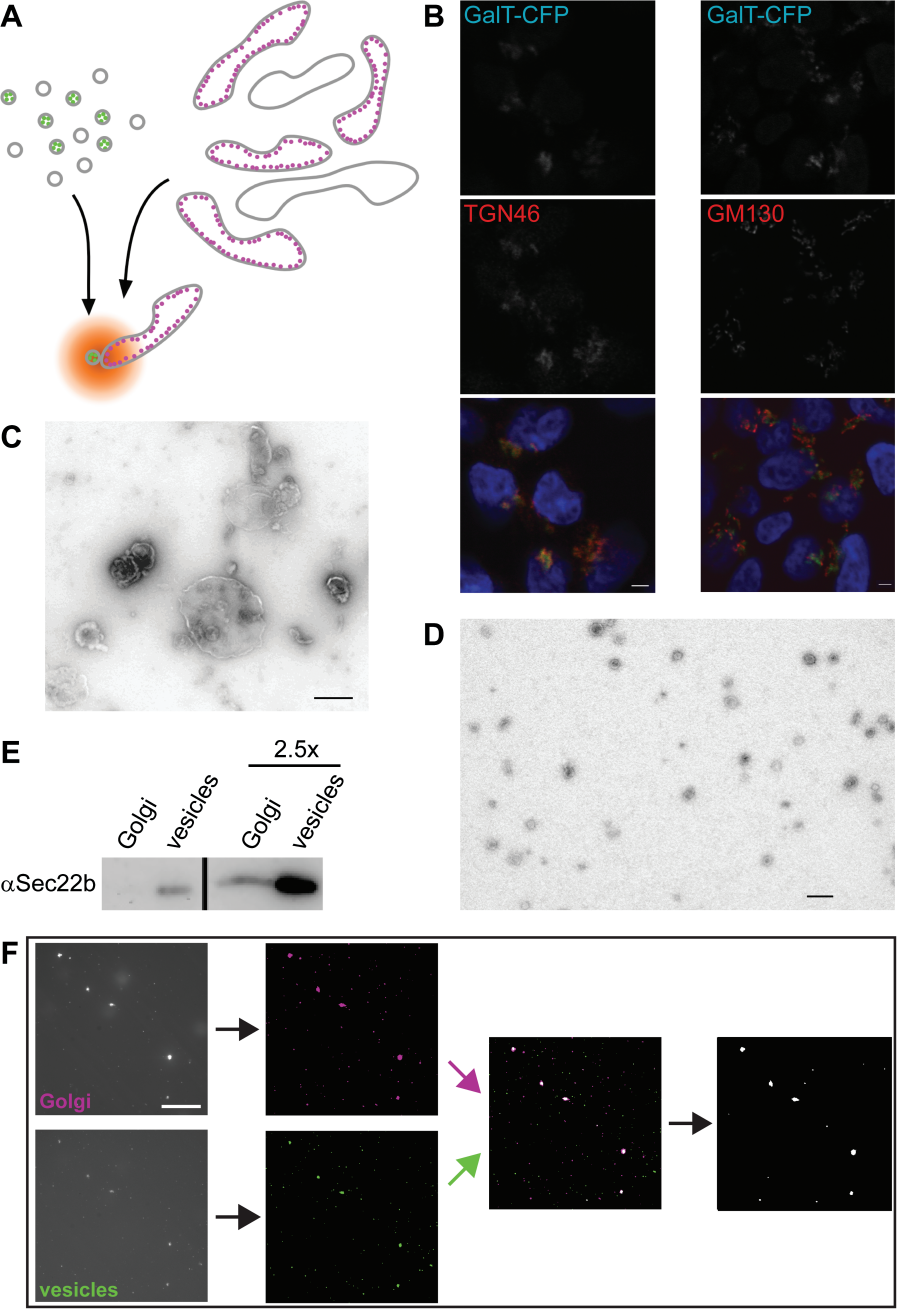
Setup of a cell-free assay using fluorescently tagged GalT as a marker. A) Schematic view of the assay in which Golgi and vesicle fractions are isolated from HEK293 cells stably expressing CFP-tagged (magenta) or YFP-tagged (green) GalT. Following their coincubation under assay conditions, colocalization is determined using fluorescence microscopy. B) Confocal fluorescent micrographs of stable GalT-CFP (green)-expressing HEK293 cells immunostained for TGN46 (left, red) or GM130 (right, red). Blue in the merge image shows DAPI staining, scale bar is 2 µm. C and D) Negatively stained electron micrographs of Golgi (C) and vesicle (D) fractions. Scale bars are 500 nm (C) and 200 nm (D). E) Immunoblot against Sec22b of equivalent Golgi and vesicle amounts. The two lanes on the right contain 2.5-fold more membrane material than the left ones. F) Typical imageJ-based data analysis workflow after image acquisition. Following noise reduction and background subtraction, binary images were overlaid and the particles containing at least one colocalizing pixel exported to be counted. For details, see *Materials and Methods*. Scale bar, 10 µm.

While cisternal maturation carries secretory cargo forward through the Golgi stack, vesicle trafficking is required for the recycling of glycosylation enzymes to their steady-state locations (28). Thus, vesicles carrying a specific enzyme are targeted to the cisterna housing that enzyme. We used this property to design a cell-free reconstitution of vesicle transport that could be used to investigate the details of vesicle tethering. The enzyme GalT was fluorescently labelled to monitor the locations of both its vesicular carrier and target compartment *in vitro* (Figure 1A). Two stable HEK293 cell lines were generated, expressing full-length GalT tagged with either eCFP or eYFP. Confocal microscopy (Figure 1B and data not shown) was used to confirm that the C-terminal fluorescent tags did not perturb the late-Golgi localization of GalT. While tagged GalT colocalized well with the *trans*-Golgi marker TGN46, it consistently localized right next to the *cis*-Golgi marker GM130 (Figure 1B). This shows that fluorescently tagged GalT localizes at the *trans*-Golgi as does the endogenous protein (27). Golgi membranes isolated by gentle cell disruption followed by sucrose gradient floatation (5) yielded 0.5–1.5 µm large membrane particles (Figure 1C). Concurrently, vesicles isolated by Optiprep gradient floatation (13) gave 50–80 nm membrane particles (Figure 1D), as expected. Isolated Golgi fractions contained 2.20 ± 0.51 x 10^7^, whereas vesicle fractions contained 2.11 ± 0.25 x 10^8^ (*n* = 4 for both) fluorescent particles per microgram of protein. The relative amount of the Golgi-vesicle marker Sec22b (29) was significantly enriched in vesicles compared with Golgi membranes, demonstrating that the isolated vesicles were not simply small Golgi fragments (Figure 1E).

In a typical 50-μL assay, Golgi and vesicle membranes containing approximately 7 and 0.7 µg protein, respectively, were used to ensure a good number of fluorescent particles during imaging. Membranes were mixed with cytosol and an energy regeneration system (5) before incubation at 37°C for 40 min. Control reactions were kept on ice, a temperature shown to inhibit membrane transport at a stage preceding vesicle tethering (9). An aliquot of each sample was then imaged using an emCCD camera, and the images processed to remove noise and overlay the two different fluorescent channels (Figure 1F). Counting of the colocalized Golgi and vesicle particles gives a measure of transport activity in the assay. For each assay, 10–15 different fields of view were imaged per sample, which usually resulted in well over 1000 counted particles for both Golgi and vesicles. At 0°C the colocalizing particles could make up between 0.5 and 2% of the total, whereas at 37°C these values ranged from 2 to about 7%. Activity is expressed as the ratio of the 37°C and 0°C results, and yields 3.16 ± 0.33 [standard error of the mean (SEM) for *n* = 8] for a full assay. This shows that physiological incubation in the presence of energy and cytosol resulted in a threefold increase of Golgi-vesicle colocalization when compared with the identical mix incubated on ice.

### Physiological relevance of the cell-free reconstitution

**Figure 2:**
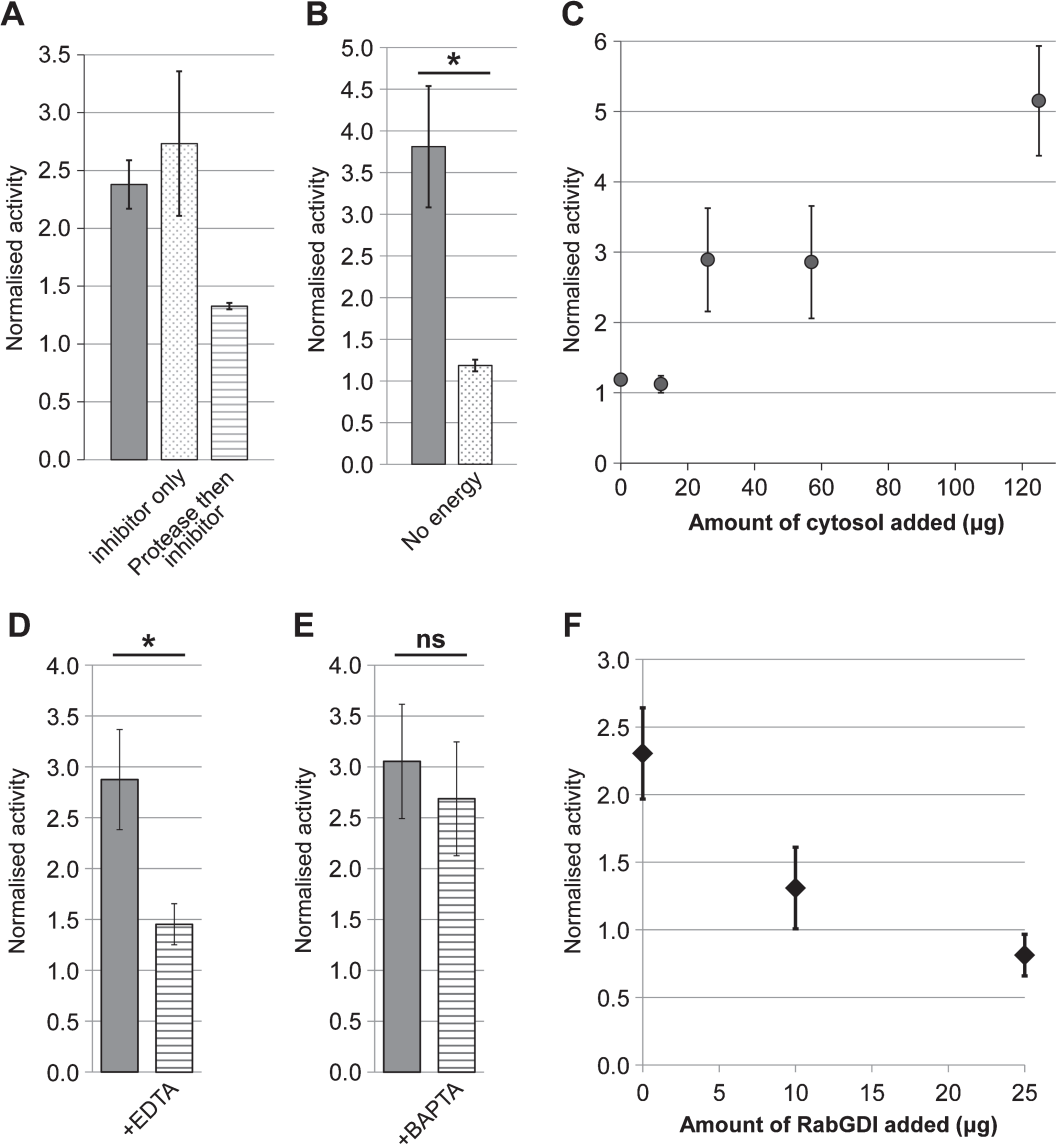
Testing the assay under physiological conditions. A) Assays in the presence of membranes, cytosol and energy were performed (grey bar) in the presence of the protease inhibitor Pefabloc (dotted bar) and in the presence of Pefabloc added following a 30-min incubation on ice with Proteinase K (striped bar). Error bars show SEM for *n* = 2. B) Assays performed in the presence of membranes and cytosol with (grey bar) or without (dotted bar) energy source. Error bars show SEM for *n* = 3. C) Assay activity in the presence of increasing amount of cytosol. Error bars show SD for *n* = 2. Note that standard assays used 57 µg cytosol to allow the addition of other factors without increasing osmolarity beyond 310 mOsm. D and E) Activity in the presence of the chelators EDTA (D, *n* = 6) or BAPTA (E, *n* = 5). Error bars represent SEM. F) Assay activity in the presence of increasing amounts of RabGDI. Full assay mix was preincubated with the indicated amount of RabGDI for 20 min on ice before addition of the energy regeneration system and normal assay incubation. Error bars represent SEM for *n* = 3 (0 and 25 µg RabGDI) or *n* = 2 (10 µg RabGDI). Note that the value of 1.0 is the activity observed in the ice-control sample. Statistical significance was determined as described in the *Materials and Methods* section, *p < 0.05; ns, not significant.

We next performed a series of control experiments to test whether the assay reconstitutes physiological membrane transport. When only the protease inhibitor Pefabloc was present during the assay, activity was unaffected, whereas incubation with the non-specific protease proteinase K for 30 min prior to inhibitor protection reduced activity to background levels (Figure 2A). This implies that transport in the assay depends on one or more intact proteins. It has been shown previously that energy is necessary for *in vitro* vesicle transport within the Golgi (13). When energy was omitted from our assay, activity fell to background levels (Figure 2B). Similarly, vesicle transport has been shown to require cytosolic proteins such as NEM-sensitive factor (NSF) and α-SNAP, particularly when the membranes are stripped of essential components by treatments such as high-salt washes (30). We found that activity became cytosol dependent when a concentrated vesicle stock was diluted at least 450-fold into the assay mix, with increasing amounts of cytosol leading to a proportional increase in activity (Figure 2C). Salt washing of vesicle membranes also caused cytosol dependence, although in this case the total activity was lower (data not shown).

Membrane transport is critically dependent on the divalent cations Mg^2+^ and Ca^2+^. Mg^2+^ is needed for the activity of nucleotide-binding proteins such as Rab GTPases (31), whereas a local increase in Ca^2+^ concentration is essential for the fusion of intra-Golgi transport vesicles (32). The generic chelator ethylenediaminetetraacetic acid (EDTA) potently inhibited activity (Figure 2D), showing that at least one cation is essential for activity in this assay. In contrast, the chelator 1,2-bis(2-aminophenoxy)ethane-*N,N,N*′,*N*′-tetraacetic acid (BAPTA), which sequesters Ca^2+^ but not Mg^2+^, did not significantly affect activity (Figure 2E). This is in line with the need for Mg^2+^ during Rab function at the tethering stage, and implies that the assay may indeed reconstitute tethering rather than fusion, because the latter would need Ca^2+^. To further investigate this point, the effect of RabGDI on activity was tested. This protein inhibits Rab GTPases by sequestering them in the cytosol. RabGDI was able to suppress activity in a dose-dependent manner (Figure 2F), further implicating the sensitivity of the assay to the vesicle tethering step.

### Dissection of tethering functions using the new assay

**Figure 3:**
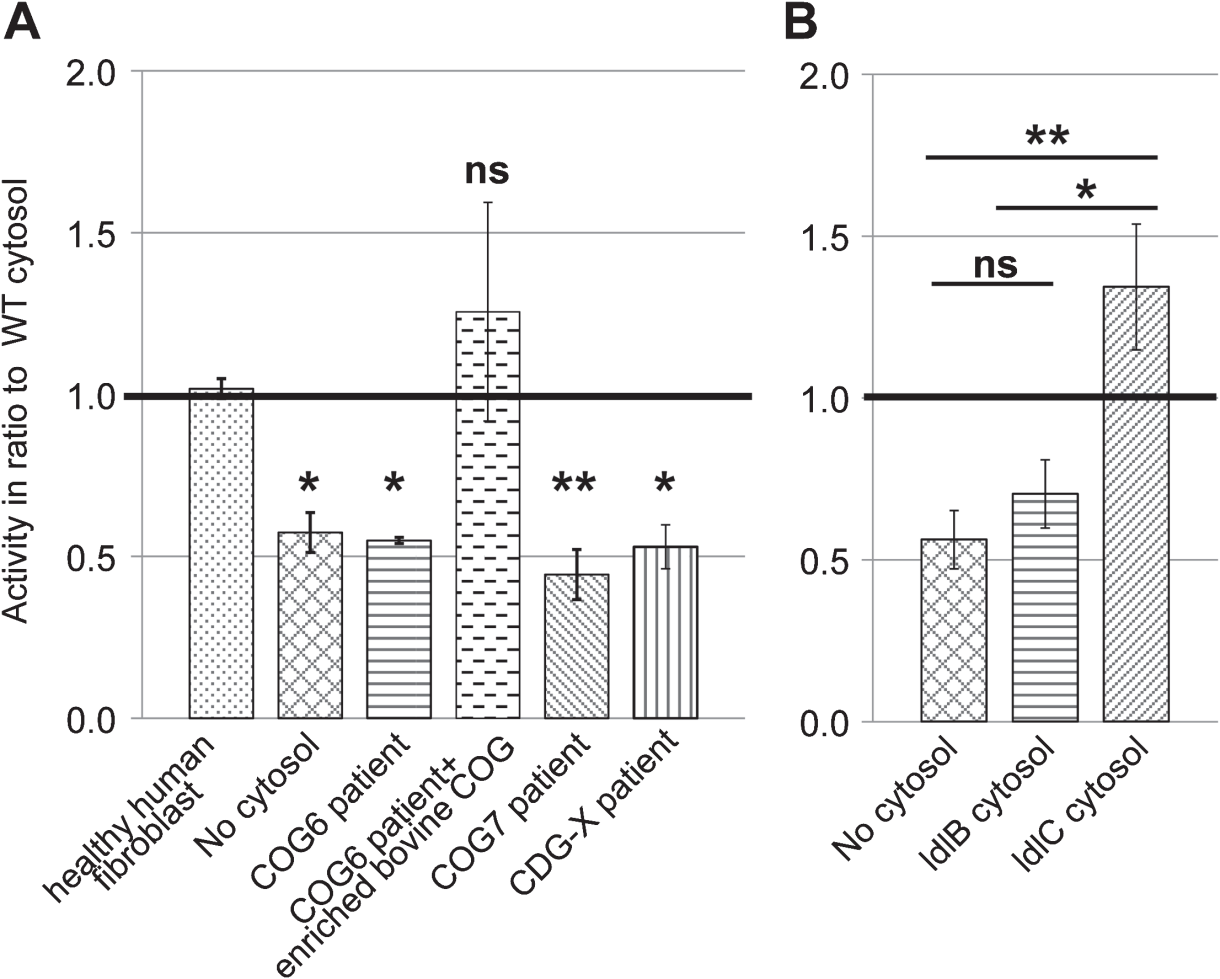
Functional distinction between lobe A and lobe B of the COG complex. A) Transport activity with cytosol from fibroblasts of COG-deficient patients as well as a novel CDG-X patient with an unknown mutation. Because of differences in cytosol and membrane concentrations between different experiments the bars show the average of the ratio between the given cytosol's activity and WT cytosol activity. The value of 1.0 therefore represents WT activity in this graph. *Escherichia coli* cytosol was used in the control without cytosol. SEM calculated for no cytosol, COG6 and COG7 *n* = 4, CDG-X *n* = 3 and complemented COG6 and human fibroblast *n* = 2. See Figure S1, Supporting Information for the individual assays used for calculating the ratios for the COG7-deficient cytosol as an example. B) Transport activity in the presence of cytosol isolated from COG mutant CHO cell lines. ldlB cells (horizontal stripes) are COG1 deficient, whereas ldlC cells (slanted stripes) are COG2 deficient. All details as in ‘A’, activities are shown in ratio to WT CHO cytosol. Error bars represent SEM for *n* = 4. Although *E. coli* cytosol was used as a protein mix in ‘A’ to suppress non-specific membrane aggregation, we found that omission of this did not cause a difference, and therefore conducted some of the experiments in ‘B’ without this additive.

Given that the assay satisfied several criteria for the physiological reconstitution of vesicle tethering, we wondered whether it could be used to assess the function of tethering factors. The COG complex was chosen for these investigations, because it is needed for all retrograde vesicle tethering reactions at the Golgi (16). Fibroblasts derived from Cog6-and Cog7-deficient patients who have defects in terminal glycosylation reactions including galactosylation (21,23) were first used. When cytosol isolated from Cog6-and Cog7-deficient fibroblasts was compared to cytosol from healthy human fibroblasts, transport activity was reduced to background levels (Figure 3A). The activity could be restored by addition of a highly enriched COG fraction purified from bovine brain to the Cog6-deficient cytosol (Figure 3A, third and fourth bars). Thus, the assay can indeed assess COG function, and is therefore suitable for the *ex vivo* analysis of COG-dependent CDG defects. To expand the potential usefulness of the assay, a hitherto uncharacterized CDG case, CDG-X, was also analysed. Serum from this patient displayed an abnormal mass spectrometric transferrin profile, including both sialylation and galactosylation defects, which was consistent with a possible defect in Golgi trafficking. Yet, no mutation in COG was found in this patient (H.H. Freeze, unpublished data). The cytosol derived from this patient lacked transport activity (Figure 3A, last bar), demonstrating that our assay is also sensitive to a CDG-derived defect that is independent of COG.

The experiments using Cog6-and Cog7-deficient cytosol suggest that lobe B of the COG complex is necessary for the tethering of GalT-containing vesicles. The two lobes have been suggested to play differential roles in the targeting of early-and late-Golgi retrograde transport vesicles, with lobe A playing a more prominent role at the early Golgi and lobe B playing a more prominent role at the late Golgi (24–26). Lobe A mutants were therefore also tested for their effect on assay activity using the Chinese hamster ovary (CHO) cell lines ldlB, which is Cog1 deficient, and ldlC, which is Cog2 deficient (14). Although the phenotypes of these cells are nearly identical (17), ldlB-and ldlC-derived cytosols behaved differently in the assay. ldlC cytosol supported assay activity consistently above the level of wild type (WT), whereas ldlB cytosol failed to support transport (Figure 3B). The primary difference between ldlB and ldlC cytosol is the ratio of lobe B and the bulk of lobe A. In the ldlC cytosol lobe B is in excess, as lobe A is absent, whereas in ldlB cytosol the Cog2–4 trimer of lobe A is in excess (33). Thus, it is tempting to speculate that this novel assay could uncover a potential new inhibitory role for lobe A in *trans*-Golgi transport that could not be detected in previous *in vivo* studies.

## Discussion

This study describes the development of a new cell-free assay reconstituting intra-Golgi transport. By several criteria, our assay reconstitutes a physiological process. First, as protease treatment eliminates activity, it is protein-dependent. This finding argues that activity does not arise from simple membrane aggregation. Activity also depends on physiological temperatures, as would be expected from a process requiring membrane fluidity, such as membrane fusion or budding, but not necessarily from membrane tethering. Although synthetic liposomes containing SNARE proteins are seen to associate at 0°C (34), endosomes tether to each other only at physiological temperatures (9). A further requirement for both endosomal transport and intra-Golgi transport *in vitro* is an energy source (5,7,9,13), which is likewise required for activity in our assay. In addition, the universal need for Rab GTPases in vesicle transport could also be recapitulated by inhibition of the activity through treatment with RabGDI.

The classic reconstitution of intra-Golgi transport relies on the enzymatic processing potential of the organelle by using the incorporation of radiolabelled sugar into a model glycoprotein (5). The new assay described here provides several advantages. First, while an enzymatic readout depends on a number of factors including the efficiency of the enzymatic reaction and the completion of other glycosylation reactions that are a prerequisite for label incorporation (35), the new assay provides a more explicit readout of tethering without a coupled enzymatic step. Successful tethering results in colocalization of the differently coloured markers allowing direct visualization. Therefore, this assay should be more useful in elucidating molecular mechanistic details of tethering, as we have started to do with the COG complex. Second, the fluorescent readout informs solely on the specific transport step involving the labelled glycosylation enzyme – in this case GalT. Conversely, the classical assay is similarly sensitive to the perturbation of all SNAREs throughout the Golgi stack without preference for a given intra-Golgi vesicle transport step (36). It should be straightforward in the future to generate fluorescently marked enzymes residing in medial and *cis*-Golgi compartments to study the different vesicle targeting requirements throughout the Golgi stack.

The usefulness of the assay is highlighted by its ability to start dissecting functions of the two lobes of COG that so far could not be directly tested. COG has been shown to stimulate the classical Golgi-transport assay, and antibodies against the lobe B subunit Cog5 inhibited the same (37). Because of the exquisite sensitivity to a specific *trans*-Golgi transport step, the fluorescence-based assay described here is capable of further dissecting COG's involvement in intra-Golgi retrograde transport. Cytosol containing reduced levels of lobe B, derived either from mutant CHO cells or from patients, cannot sustain transport activity when lobe A levels are unchanged (21,23,33). However, the same amount of lobe B in ldlC cells is perfectly competent for transport, albeit its level is still only 10% of the WT steady-state level, when lobe A is eliminated (33). The property of lobe B to promote transport by itself at the *trans*-Golgi is consistent with previous *in vivo* reports of lobe B function (24,26). Yet, a possible inhibitory effect of lobe A is a novel suggestion for COG function, which will have to be further tested in the future.

The sensitivity of the assay to *trans*-Golgi transport, which relies on the choice of GalT as a marker, is essential for the shown functional dissection of COG. At the same time this activity opens up new possibilities for the characterization of novel CDG cases. COG-dependent CDGs can be identified by a characteristic delay of the brefeldin A-induced relocalization of enzymes to the endoplasmic reticulum (38). Yet, it is likely that other types of trafficking-related CDGs do not show this characteristic; indeed, the CDG-X case used in this study does not present with an unambiguous delay in the effects of brefeldin A (B.G. Ng and H.H. Freeze, unpublished observation). Most CDG mutations affecting Golgi trafficking are hypomorphs in which the glycosylation phenotype is restricted to *trans*-Golgi defects, such as galactosylation and sialylation problems (22,39). This is likely because similarly pleiotropic glycan aberrations earlier in the glycosylation pathway would not survive embryonic development. For this reason an assay that is a sensitive measure of vesicle transport at the *trans*-Golgi will be useful for the cellular characterization of many new CDG cases involving Golgi-organization defects, as we have started to do with the described CDG-X case. This is another advantage compared with the classical Golgi-transport assay, which used GlcNAc incorporation as the readout (5), and thus a glycosylation step that is too early for the successful investigation of most trafficking-related CDGs. For some of these CDGs though, a sialyltransferase-based assay, which can be easily constructed based on our GalT-based template, may be even more appropriate. Having a new assay opens up the possibilities of complementation and mechanistic studies that will help better understand these diseases.

## Materials and Methods

### Materials

All chemicals were from Sigma and cell culture reagents from Invitrogen, unless otherwise stated. Affinity-purified rabbit α-Sec22b (used at 1:1000) was a kind gift from J. Hay (U. Montana). All procedures except for the appropriate assay incubations were performed at 4°C or on ice, unless otherwise stated. Borosilicate microscope slides and cover slips were from Menzel Gläser, and were cleaned before use by soaking in several changes of 3% decon90 (Decon Laboratories Limited) for a day followed by thorough rinsing in water and drying in a 60°C oven. Five-microlitre dry volume of 5-µm silica beads (Bangs Laboratories) was suspended in 500 μL of 150 mM KCl, 10 mM HEPES, pH 7.2, by vigorous vortexing and 30-second agitation in a sonicating water bath. Beads were used at a density to give one to two beads per field of view in the assay.

### Plasmids

Full-length GalT cDNA (kind gift from M. Fukuda) was cloned into a pCR3.1+ vector modified to contain an HA or myc tag followed by a seven-amino acid linker and an eCFP or eYFP tag, all downstream of the GalT gene.

### Cell culture

HEK293 cells and human-derived fibroblasts were cultured in DMEM supplemented with 10% FBS and 2 mM GlutaMAX-I. HEK293 cell clones stably expressing GalT-XFP constructs were selected in complete media in the presence of 0.8 mg/mL Geneticin. WT and mutant CHO cells were cultured in Ham's F-12 Nutrient Mixture supplemented with 5% FBS and 2 mM GlutaMAX-I.

### Isolation of membranes and cytosol

Golgi membranes were isolated as described (5). Briefly, cells from two confluent T175 flasks were harvested and washed in 0.25 M sucrose, 10 mM HEPES, pH 7.4. The cell pellet was resuspended in four times the pellet volume using the same buffer. The suspension was treated with 25 strokes in a 1-mL tight fitting Dounce homogenizer. The homogenate was adjusted to 1.4 M sucrose by adding 2.3 M buffered sucrose and overlaid with 1.2 and 0.8 M buffered sucrose layers in an SW41 tube. Following centrifugation for 80 min at 39 000 rpm the turbid band containing Golgi membranes at the 1.2/0.8 M sucrose interface was then flash frozen in liquid nitrogen in small aliquots and stored at −80°C.

Vesicles were isolated as described (13). Briefly, cells harvested from four confluent T175 flasks were washed in PBS and then 0.2 M sucrose, 10 mM Tris, pH 7.2. The pellet was resuspended in four times the pellet volume using the 0.2 M sucrose buffer, flash frozen in liquid nitrogen and then gradually thawed in a water bath at 21°C. Cell debris was removed by centrifugation twice each at 1000 and 20 000 × ***g*** collecting vesicles in the supernatant. Vesicles were then pelleted by centrifugation at 55 000 rpm for 45 min in a TLA 100.3 rotor (Beckman Coulter). The pellet was resuspended in KHM buffer [150 mM KCl, 2.5 mM Mg(OAc)_2_ and 10 mM HEPES, pH 7.2] to a volume of 320 μL, mixed with 480 μL of 50% Optiprep in HM buffer (KHM without KCl) and the mixture overlaid with 800 μL 25% and 400 μL 10% Optiprep in KHM in a TLS-55 tube (Beckman Coulter). Vesicles that floated to the 10/25% interface by spinning at 55 000 rpm for 3 h 10 min in a TLS-55 rotor were harvested by pipetting, aliquots flash frozen in liquid nitrogen and stored at −80°C. To generate cytosol-dependent vesicles the amount of pellet resuspended before floatation was scaled up sevenfold to yield a sevenfold concentrated vesicle stock.

Cytosol preparation was adapted from (5). For HEK293 cytosol, cells grown to confluency in four T175 flasks were mechanically dislodged in their growth media, then washed twice and resuspended in 1.5 mL 0.25 M sucrose, 10 mM HEPES, pH 7.4 and 1 mM DTT. The suspension was lysed by 30 strokes in a 1-mL tight fitting Dounce homogenizer and centrifuged twice at 60 000 rpm in a TLA 100.3 ultracentrifuge tube for 45 min. The supernatant was desalted over a PD-10 column (GE Healthcare) equilibrated with cytosol buffer (100 mM KCl, 1 mM DTT and 10 mM HEPES, pH 7.2), aliquoted, flash frozen in liquid nitrogen and stored at −80°C. For the isolation of CHO and human fibroblast cytosols about 7 × 10^6^ cells were harvested using a cell scraper.

Recombinant histidine-tagged RabGDI was purified using Ni-NTA followed by MonoQ ion-exchange chromatography as described (40). The purified protein was concentrated to 10 mg/mL, dialysed into 7.5 mM HEPES, 75 mM KCl, 0.5 mM DTT, pH 7.2, and filtered before use in the assay.

Partial purification of the COG complex through ammonium sulphate precipitation and butyl sepharose chromatography as described (14) was followed by TSK4000SW (Toso Haas) gel filtration chromatography (in cytosol buffer) that results in a highly enriched COG fraction (14). Enriched COG was concentrated to 1.4 mg/mL and filtered before use in the assay. This fraction is only active for about 10 days at 4°C.

### Immunofluorescence and confocal microscopy

Cells grown on cover slips coated with poly-d-lysine (BD Biosciences) were fixed with paraformaldehyde, permeabilized and stained with α-TGN46 polyclonal antibody (Millipore, 1:600), α-GM130 monoclonal antibody (BD Biosciences, 1:500) followed by Alexa-568-conjugated goat α-rabbit (for TGN46) or α-mouse (for GM130) antibody (Life Technologies, 1:400) and DAPI (1:2000). Cover slips were mounted using Aqua-Poly/Mount (Polysciences) and imaged using an inverted Zeiss confocal microscope with a 63× objective.

### Electron microscopy

Four-microlitre diluted membrane particles were placed onto a copper grid (square 200 mesh, Agar Scientific) and left for 5 min. Golgi membranes were briefly fixed with glutaraldehyde prior to dilution. The grid was rinsed with water and then negatively stained for 5 min with 1% uranyl acetate. Stained grids were viewed in a Tecnai G2 12 BioTWIN transmission electron microscope (FEI) at 120 kV.

### Cell-free assay

A total of 3.8 μL of Golgi membranes, 0.8 μL vesicle membranes (diluted sevenfold with KHM in the case of the more concentrated stock) and 57 µg cytosol were mixed in a final volume of 50 μL containing 150 U/mL creatine phosphokinase, 1 mM GTP, 0.5 mM ATP, 20 mM creatine phosphate, 30 mM HEPES, 30 mM KCl, 66 mM sucrose, 2 mM MgCl_2_ and 0.3 mM DTT, pH 7.4. Combined with the sucrose derived from the Golgi membrane aliquot the components in the assay mix gave an osmolarity close to 310 mOsm, which is the osmolarity of tissue culture media. After thorough mixing, 25 μL was incubated for 40 min at 37°C, while the remaining 25 μL was kept on ice. Both samples were kept in the dark. Reactions were stopped by placing samples on ice, and following the addition of a 1-μL suspension of 5-µm beads, 3 μL of each sample was mounted using 22-mm square cover slips and then fully sealed with clear nail varnish. Slides were stored at 4°C until imaging.

To generate the assay mix, very small amounts of occasionally viscous stock solutions had to be handled, resulting in substantial variation in background activity. The effect of this variation could be minimized by splitting an assay after mixing to generate the 0°C and 37°C samples, and reporting the activity as a ratio of the 37°C and 0°C activities.

Images were collected using an Evolve 512 emCCD camera (Photometrics) attached to a Zeiss Axiovert 200M fully motorized inverted microscope (Carl Zeiss) with a Zeiss Plan-Apochromat 63×/1.40 Oil DIC objective and an eCFP/eYFP filter set (Chroma Technology). Illumination was provided by an X-Cite 120Q light source (Lumen Dynamics Group). The adjustable iris of the X-Cite was opened 75%. The analogue-to-digital gain was 3 and image quality was 16-bit for all images. Before capturing an image the microscope was focussed onto the vesicles using a 100-millisecond exposure time with an EM gain of 200 and 2 × 2 binning. Vesicles were then imaged using a 5-second exposure, EM gain of 23, while Golgi with an exposure of 3 seconds and EM gain of 6, both without binning. EM gain parameters had to be slightly altered as the illumination source slightly decayed over a period of months. At least 10 fields of view were imaged for each sample.

### Data analysis

Image processing and analysis was performed using imageJ. Following their conversion to 8-bit, noise in the images was reduced using the ‘subtract background’ routine with a rolling ball radius of 2.0 pixels and a sliding paraboloid. The threshold was then set to ‘triangle dark’ for Golgi images and the same for vesicle images except that the upper threshold was multiplied by 1.4. Binary images were generated from the selections, and the ‘lookup table’ for the binary image changed to ‘red’ for Golgi and ‘green’ for vesicles. After converting the Golgi (red) and vesicle (green) binary images to RGB they were merged, and the number of yellow (colocalizing) as well as all Golgi and vesicle particles counted. Using Excel, the number of colocalized particles was expressed as a percentage of the total number of particles (Golgi + vesicles + colocalized), which is a measure of activity in the sample. The activity of each sample measured at 37°C was normalized to the activity of the same sample at 0°C. For more complex series of data using cytosols from mutant cells the normalized activities are presented as a ratio of the mutant and WT activities (see Figure 3). Error bars were calculated as standard error of the mean for averaged experiments carried out on separate occasions.

### Statistical analysis

For pairwise comparison of data (Figure 2) a one-tailed Student's *t*-test (unpaired, unequal variance) was performed with the hypothesis that non-standard assay mixtures will reduce activity.

For data presented in Figure 3 a one-way anova was used either with a Dunnett *post hoc* test (for Figure 3A) using WT fibroblasts as the control or with a Tukey–Kramer *post hoc* test (Figure 3B). Significant differences are indicated as *p < 0.05 and **p < 0.01.

## Supporting Information

Figure S1 Four separate experiments containing different batches of the same components. For details, see Figure 3A.
