# Supplementary material for: Dissecting Functions of the Conserved Oligomeric Golgi Tethering Complex Using a Cell-Free Assay
Source: Traffic. 2013 Oct 31;15(1):12–21. doi: 10.1111/tra.12128 (PMC3892563; doi:10.1111/tra.12128)
Supplement: Figure S1 — Four separate experiments containing different batches of the same components. For details, see Figure 3A. [file tra0015-0012-sd1.pdf]

Cottam et al Supplementary Figure 1

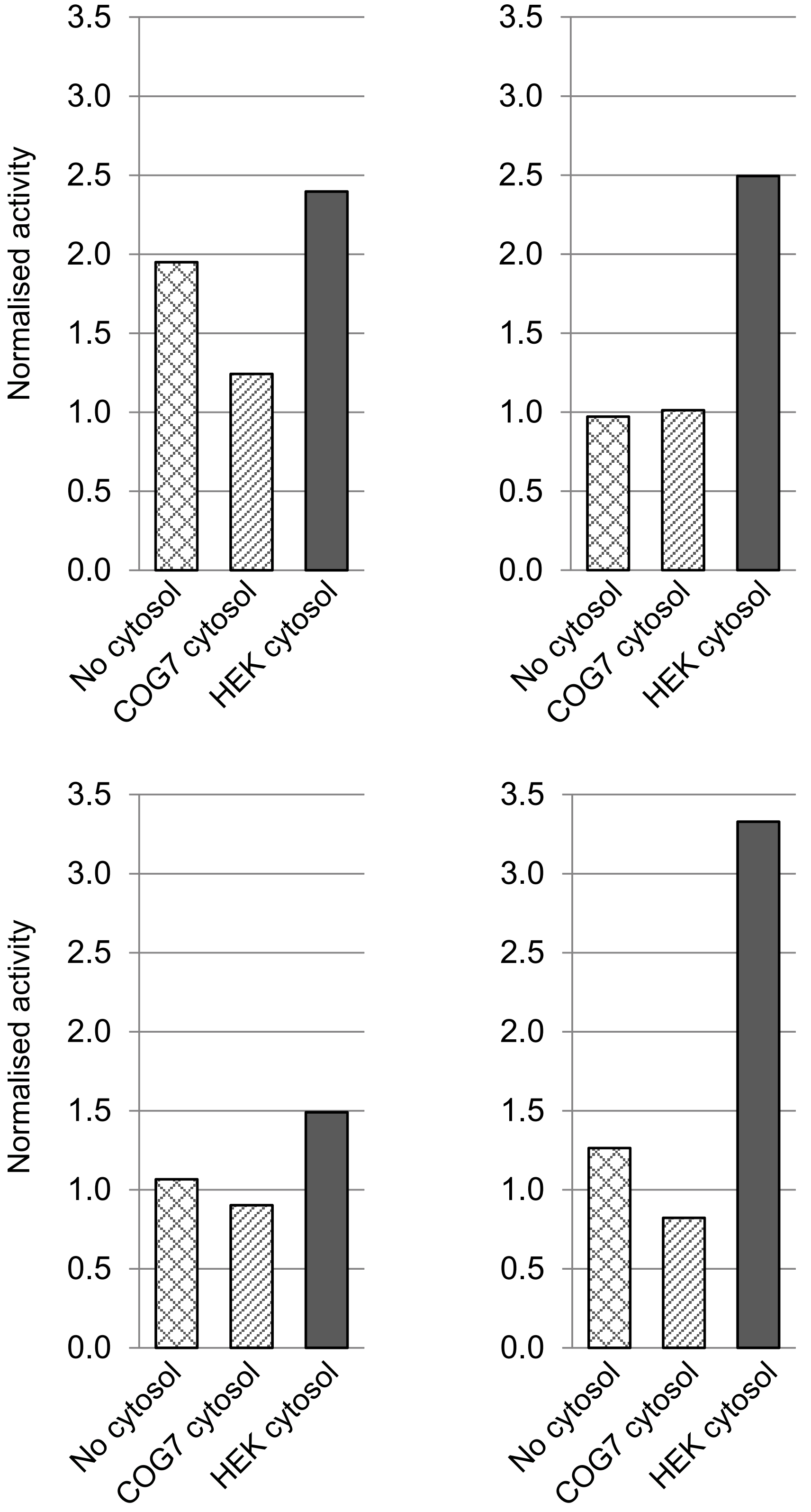

Four separate experiments containing different batches of the same components. For details see Fig. 3A.
